# Stratify or adjust? Dealing with multiple populations when evaluating rare variants

**DOI:** 10.1186/1753-6561-5-S9-S101

**Published:** 2011-11-29

**Authors:** Robert C Culverhouse, Anthony L Hinrichs, Brian K Suarez

**Affiliations:** 1 Department of Medicine, Washington University School of Medicine, 660 South Euclid Avenue, Saint Louis, MO 63110, USA; 2 Division of Biostatistics, Washington University School of Medicine, 660 South Euclid Avenue, Saint Louis, MO 63110, USA; 3 Department of Psychiatry, Washington University School of Medicine, 660 South Euclid Avenue, Saint Louis, MO 63110, USA; 4 Department of Genetics, Washington University School of Medicine, 660 South Euclid Avenue, Saint Louis, MO 63110, USA

## Abstract

The unrelated individuals sample from Genetic Analysis Workshop 17 consists of a small number of subjects from eight population samples and genetic data composed mostly of rare variants. We compare two simple approaches to collapsing rare variants within genes for their utility in identifying genes that affect phenotype. We also compare results from stratified analyses to those from a pooled analysis that uses ethnicity as a covariate. We found that the two collapsing approaches were similarly effective in identifying genes that contain causative variants in these data. However, including population as a covariate was not an effective substitute for analyzing the subpopulations separately when only one subpopulation contained a rare variant linked to the phenotype.

## Background

The Genetic Analysis Workshop 17 (GAW17) unrelated individuals sample is derived from the pilot3 study of the 1000 Genomes Project (http://www.1000genomes.org) and consists of genotypes of 697 subjects drawn from 8 populations. Of the 24,487 exomic single-nucleotide polymorphisms (SNPs) in the data, 9,433 (38.5%) occur only once in a single individual and 18,131 (74.0%) occur with less than 1% minor allele frequency (MAF). Phenotypes provided include sex, age, smoking (yes/no), ethnic population, three quantitative traits (Q1, Q2, and Q4), and the dichotomous trait Affected. A single genetic model based on additive genetic effects was used for all subjects. For a full description of the data simulation, see Almasy et al. [1]. As a result of these conditions, we took a gene-centric approach to our analysis. We had two goals: (1) to determine whether any genes that contribute to the generating model could be detected using only rare variants in these extremely sparse data and (2) to determine whether population stratification would be better dealt with using stratified analyses or simply including population as a covariate.

We were blind to the generating model before the GAW17 meeting so that our analyses would not be biased by knowledge of the true model. The blind was broken at the GAW17 meeting, and our knowledge of the generating model was used for the evaluation of methods discussed in this paper.

## Methods

Our analyses were based on 2,448 genes, each having at least 1 rare SNP (minor allele frequency [MAF] < 0.01) from the total 3,205 genes included in the data. This arbitrary threshold was chosen as a compromise between what is typically considered common (MAF ≥ 0.05) and the fact that the sample size in the provided data was modest. After inspecting the generating model, we discovered that 5 out of 39 causative variants for Q1 fell between these two thresholds, as did 2 of the 51 variants for affection status. We used a regression framework to examine the quantitative trait Q1 and the dichotomous trait Affected.

### Collapsing rare variants

We generated two genetic variables based on related collapsing approaches. The first variable was simply a count of how many rare alleles an individual carried for a particular gene. The second variable was dichotomous, indicating whether or not an individual carried at least one rare allele in a particular gene. Both of these collapsing approaches were previously discussed by Li and Leal [2] as part of a more sophisticated analytic approach that incorporates both rare and common variants.

### Using multiple data replicates

Because of the sparseness of the information in the unrelated individuals sample, we believed that a single data replicate would likely be underpowered for this analysis. Each replicate contains exactly the same genotypes, making most approaches to combining information from multiple replicates prone to spurious associations. The focus on rare variants in this analysis exacerbates this problem. We chose to perform a meta-analysis of the multiple replicates. For these particular data, this approach provides a scalability feature that allows easy comparisons of differing sample sizes. For the full data, we examined single replicates, and meta-analyzed sequential groups of 10 replicates each (e.g., replicates 1–10, 11–20, etc.) and the first 50 replicates. For the much smaller subpopulation samples, we meta-analyzed sequential groups of 10 replicates each and the first 50 replicates.

An initial examination of the quantitative traits indicated that Q4 was largely determined by the covariates Sex, Age, and Smoking. This made Q4 a good candidate to use to evaluate the extent to which combining multiple replicates would lead to entirely extraneous false positives. We therefore performed the same regression analyses and meta-analyses on Q4 as we did for Q1. The use of Q4 as a negative control for false positives allowed us to evaluate the chances of the single set of genotypes giving rise to entirely spurious signals. We note that the use of a negative control lets us evaluate only the extent to which entirely spurious signals might arise from the use of multiple copies of the same genotypes. However, this approach cannot provide an estimate of the extent to which small spurious signals, resulting from such things as rare variants in individuals with extreme phenotypes or modest correlations between a causative gene and a null gene, might be amplified when using multiple replicates.

### Population stratification

We evaluated two methods for dealing with population stratification: (1) analyzing the strata in separate analyses and (2) pooling data from all strata, using population as a covariate. Through the use of meta-analyses of varying numbers of data replicates, we could also compare results from similarly sized single-population analyses and pan-population analyses.

### Analyses

Our analyses for Q1 were based on linear regression using Sex, Age, Smoking, and Population as covariates and one of our two ways of coding the genes (quantitative count or dichotomous indicator) as the predictor of interest. We noted that the dichotomous Affected phenotype was highly correlated with the quantitative traits. As a result, we believed that our top signals from a straightforward analysis of this trait might reveal only genes associated with Q1 or Q2. Therefore, to detect genes associated directly with the Affected phenotype, in our logistic regression analyses of affection status we included Q1, Q2, and Q4 as well as Sex, Age, Population, and Smoking as covariates.

We considered the possibility that if a causative variant were found in only one subpopulation, it might be advantageous to analyze that subpopulation separately. To evaluate this possibility, we conducted a second set of analyses, performed separately on each genetically distinct subpopulation. To determine whether any of the samples could be pooled, we first performed an EIGENSTRAT analysis [3]. The results suggested that the Asian samples (Han Chinese, Denver Chinese, and Japanese) could be pooled, as could the European samples (European-descended Utah population [CEPH] and Tuscans). The differences between the two African populations were greater than any of the other groups but were still modest. As a consequence, we decided to separate them. A detailed plot of the two African populations can be found in Hinrichs et al. [4].

Finally, we note that one of the CEPH subjects (NA7347) was an extreme outlier for Q1 across most of the data replicates. This subject was excluded from our analyses.

Our multireplicate analysis plan proceeded as follows: First, we analyzed *k* individual data replicates and retained the *p*-values. Then we performed meta-analyses using Fisher’s statistic,(1)

which under the null hypothesis would be expected to have a chi-square distribution with 2*k* degrees of freedom (df). The nonindependence of the genotypes between the data replicates violates the distributional assumptions. However, our chief goal was to determine whether any of the true signals would stand out from the bulk of the noise in this sparse data.

Because of the large number of tests performed, we used 10^−6^ as our threshold for statistical significance. All statistical analyses were performed in SAS [5].

## Results

### Q1 results

**Figure 1:**
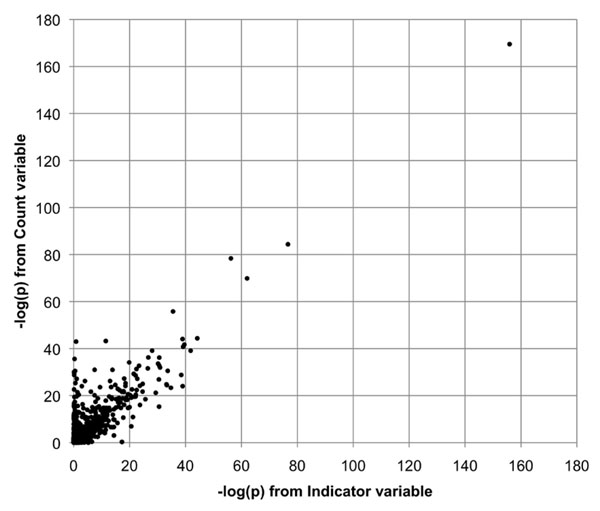
**Comparison of Q1 results from two gene-wise collapsing methods for rare variants**. Each data point represents the results of meta-analysis of a single gene using the first 50 data replicates. The horizontal position is −log(*p*) from the analysis based on the Indicator variable; the vertical position is based on the Count variable. The correlation between the two values is 0.92. If we eliminate the top four values, the correlation is still 0.86.

Table 1 provides a summary of the results of the analyses of Q1 using single data replicates, using Population as a covariate and both collapsing variables. We note that the same four genes passed our significance threshold for both of the collapsing variables (Count and Indicator) and had similar *p*-values. The high degree of similarity between the two collapsing approaches held true throughout our analyses. As an example, Figure 1 illustrates the relationship between the −log(*p*) values for the genes in a meta-analysis of Q1 using the first 50 replicates. Each point represents the results of meta-analysis of a single gene. The horizontal position is −log(*p*) from the analysis based on the Indicator variable; the vertical position is −log(*p*) from the Count variable. The correlation between the values from the two collapsing methods is 0.92. If we eliminate the top four outlying values, the remaining values still have a correlation of 0.85. For this reason, we report only the results from the Count variable in the remainder of this paper.

**Table 1:** Q1 single replicate results for combined populations (200 replicates, *N* = 697 each)

		Count (quantitative)		Indicator (dichotomous)	
		
Gene	Chromosome	Median (*p*)	Min, max	Median (*p*)	Min, max
*FLT1*	13	3.1 × 10^−19^	1.1 × 10^−29^, 5.0 × 10^−10^	9.7 × 10^−19^	9.2 × 10^−30^, 1.2 × 10^−10^
*ZNF605*	12	7.2 × 10^−10^	5.0 × 10^−19^, 1.8 × 10^−4^	7.2 × 10^−10^	5.0 × 10^−19^, 1.8 × 10^−4^
*TAS2R48*	12	8.1 × 10^−9^	9.4 × 10^−16^, 2.0 × 10^−2^	3.0 × 10^−8^	4.8 × 10^−15^, 2.3 × 10^−2^
*ZNF84*	12	1.4 × 10^−8^	8.5 × 10^−18^, 1.7 × 10^−3^	1.4 × 10^−9^	8.5 × 10^−18^, 1.7 × 10^−3^

Linear regression results from using two collapsing approaches for rare variants in a gene. Analyses used population as a covariate. The median, minimum (min), and maximum (max) *p*-value for each gene that passed our threshold of 10^−6^ for at least 50% of the replicates are listed.

A stratified analysis of the individual populations did not have any results that passed our significance threshold. However, the European sample (CEPH and Tuscan sample combined) had *FLT1* as its top result, with *p* < 10^−5^. The inability of any of these samples to pass the significance threshold was expected because of the small sample size. Results from meta-analyses of 10 replicates at a time are found in Table 2. In each case the top signal passing the significance threshold is a gene modeled as contributing to the phenotype. In addition, although the top signal from the Luhya sample, *VEGFA*, did not quite pass the significance threshold (*N* = 1,080), it was a true positive, and the next ranked gene was three orders of magnitude less significant. This signal was consistent throughout the replicates and would become significant when more replicates were added to the meta-analysis. In contrast, we note that *VEGFA* was ranked 406th in the meta-analysis results for the combined population sample (*N* = 6,970) with median *p* = 4.6 × 10^−4^.

**Table 2:** Q1 subpopulation results (20 meta-analyses, 10 replicates each)

Subpopulation	*N*	Gene	Chromosome	Median (*p*)	Min, max
Europe^a^	1,560	*FLT1*	13	1.0 × 10^−45^	1.1 × 10^−52^, 4.6 × 10^−37^
		*KDR*	4	1.9 × 10^−41^	8.7 × 10^−58^, 1.0 × 10^−32^
Asia^b^	3,210				
Yoruba^c^	1,120	FLT1	13	9.5 × 10^−17^	9.3 × 10^−25^, 4.6 × 10^−13^
Luhya^d^	1,080	*VEGFA*	6	4.2 × 10^−6^	1.7 × 10^−14^, 1.7 × 10^−2^

Linear regression results using the Count coding for rare variants in a gene. The median, minimum (min), and maximum (max) *p*-value for the top causative genes that passed our threshold of 10^−6^ for at least 50% of the replicates are listed, plus the top result from the Luhya sample, which does not quite meet the threshold.

^a^ There were 78 lower ranked false positives (*p* < 10^−6^). The highest ranked false positive had *p* < 10^−40^.

^b^ There were no genes that passed the significance threshold of 10^−6^. Top gene had median *p* = 0.13.

^c^ There were 23 lower ranked false positives (i.e., *p* < 10^−6^). The highest ranked had *p* > 10^−14^.

^d^ No genes passed the significance threshold of 10^−6^ in at least 50% of the replicates. The second ranked gene had *p* > 10^−3^.

### Q4 results

In contrast, our analysis of the negative control phenotype (Q4) resulted in no gene with a median *p* < 0.05 in the combined population sample or any of the subpopulations. Furthermore, in none of our meta-analyses, even including all 200 data replicates, did we achieve a *p*-value that passed our significance threshold (10^−6^) either in the combined data or in any subpopulation.

### Affected phenotype results

For the combined population data no gene was significant in the individual data replicates. Only one gene, *PRKCA* on chromosome 17, was significant in meta-analyses of 10 replicates (*N* = 6,970, median *p* = 2.1 × 10^−9^; range of *p*, 9.0 × 10^−13^ to 3.4 × 10^−6^). *PRKCA* was a causative gene in the model. Another causative gene, *PIK3C2B*, was ranked 5 out of 2,448 in the meta-analyses of 10 replicates. However, it would not reach significance unless all 200 replicates were meta-analyzed.

In the stratified analyses none of the subpopulation samples passed the significance threshold, in single replicate analyses or in a meta-analysis of 10 replicates. However, if 50 replicates were meta-analyzed, *PRKCA* became significant in the Asian sample (*N* = 16,050, *p* = 3.4 × 10^−11^) and in the Yoruba sample (*N* = 5,600, *p* = 5.6 × 10^−9^) and was trending toward significance in the Luhya sample (*N* = 5,400, *p* = 4.3 × 10^−5^). This signal was not seen in the combined European sample (*N* = 7,500, *p* = 0.35).

## Discussion

Clearly, some contributing genes (*FLT1*, *VEGFA*, *PRKCA*) could be detected by an examination of rare variants in the unrelated subjects. However, it turned out that our second goal (determining whether stratified analyses or a population covariate would be more effective in dealing with population stratification in these data) gave rise to the most interesting results.

*FLT1* contained multiple rare variants with large effect on Q1. The signals from this gene were strong in the European and Yoruba populations and present in the Asian populations. (The rare variants in *FLT1* were not significant in the Luhya sample, even if 200 replicates were meta-analyzed.) Because the signal was present in subsamples representing more than 84% of the data, pooling the data and using population as a covariate maximized power.

In contrast, *VEGFA*, found in an analysis of the Luhya sample, was not near the top of the list in the combined analysis. It was not until we meta-analyzed 50 replicates of the full data (total *N* = 34,850) that this gene surpassed the 10^−6^ significance threshold (*p* = 1.4 × 10^−14^). In contrast, meta-analysis of the first 50 replicates of the Luhya subjects alone (total *N* = 5,400) resulted in an extremely low *p*-value (*p* = 2.1 × 10^−94^). This is because the rare variant for *VEGFA* is private to the Luhya population. As a result, including samples from other populations merely introduces noise into the signal. It is interesting to note that *VEGFA* corresponds to the highest linkage signal found in a linkage analysis of the family data [4].

We note that the phenotypes were modeled identically for the different populations. As a consequence, one might have believed a priori that a combined analysis (perhaps not even using population as a covariate) might be the most powerful approach. However, as illustrated by the results for *VEGFA*, this need not be the case. This suggests that it might be worthwhile to analyze multipopulation data both ways (stratified and adjusted), despite the multiple testing penalty.

In these data we also found that the two tested approaches to collapsing performed similarly, particularly for the top signals. This simply suggests that in these data the outliers in phenotype were not also outliers in terms of the count variable for any genes. Clearly, this cannot be generalized to other genetic models.

Finally, although our top signal in each analysis result was a true signal, there were many more highly significant false positives than we would have expected. We learned from the analysis of Q4 that it is unlikely that these spurious results were a completely random effect of using multiple replicates of the same genotypes. Two other possible causes come to mind. First, rare variants carried by individuals with extreme phenotypes could give rise to such results. We tested this idea by performing some analyses that included the individual from the CEPH sample (NA7347) who had an extreme Q1 value (>5 standard deviations above the mean) in nearly every replicate. We found that multiple genes that were not included in the model but for which this individual was the only carrier of rare variants became significant. Second, the signals could actually be in the data, although they were not included in the generating model. We note that many false positives in these data have been reported as consistently arising under a variety of analysis methods. For a detailed discussion of this aspect of the data, see Luedtke et al. [6].

## Conclusions

In our analyses we found that in the GAW17 data, the two collapsing methods produced similar results. More important, these analyses showed that even with the identical genetic model applied to multiple subpopulations, sample size is not the only factor that determines power. If rare causative variants are private to a subpopulation, stratified analysis might be more powerful than a combined analysis, despite a considerable decrease in sample size.

## Competing interests

The authors declare that there are no competing interests.

## Authors’ contributions

RCC conceived of the study, participated in its design, performed the main statistical analysis, and drafted the manuscript. ALH participated in the design of the study and performed the EIGENSTRAT analyses. BKS participated in the design of the study and helped to draft the manuscript. All authors read and approved the final manuscript.
